# Ion-Assisted Nanoscale Material Engineering in Atomic Layers

**DOI:** 10.1021/acs.nanolett.5c02040

**Published:** 2025-06-13

**Authors:** Hossein Taghinejad, Mohammad Taghinejad, Sajjad Abdollahramezani, Qitong Li, Eric V. Woods, Mengkun Tian, Ali A. Eftekhar, Yuanqi Lyu, Xiang Zhang, Pulickel M. Ajayan, Wenshan Cai, Mark L. Brongersma, James G. Analytis, Ali Adibi

**Affiliations:** † Department of Physics, 1438 University of California, Berkeley, California 94720, United States; ‡ School of Electrical and Computer Engineering, 1372 Georgia Institute of Technology, Atlanta, Georgia 30332, United States; § Kavli Energy NanoSciences Institute, University of California, Berkeley, California 94720, United States; ∥ School of Materials Science and Engineering, 6429 Stanford University, Stanford, California 94305, United States; ⊥ Geballe Laboratory for Advanced Materials, 6429 Stanford University, Stanford, California 94305, United States; # Institute of Electronics and Nanotechnology, 1372 Georgia Institute of Technology, Atlanta, Georgia 30332, United States; ¶ 28272 Max Planck Institute for Iron Research, Max-Planck-Straße1, Düsseldorf 40237, Germany; ∇ School of Materials Science and Nanoengineering, 3990 Rice University, Houston, Texas 77005, United States; ○ Materials Sciences Division, Lawrence Berkeley National Laboratory, Berkeley, California 94720, United States

**Keywords:** 2D materials, heterostructures, optoelectronics, atomic defects, focused ion beam

## Abstract

Achieving deterministic control over the properties of low-dimensional materials with nanoscale precision is a long-sought goal. Mastering this capability has a transformative effect on the design of multifunctional electrical and optical devices. Here, we present an ion-assisted synthetic technique that enables precise control over the material composition and energy landscape of two-dimensional (2D) atomic crystals. Our method transforms binary transition-metal dichalcogenides, like MoSe_2_, into ternary MoS_2α_Se_2(1−α)_ alloys with systematically adjustable compositions, α. By piecewise assembly of the lateral, compositionally modulated MoS_2α_Se_2(1−α)_ segments within 2D atomic layers, we present a synthetic pathway toward the realization of multicompositional designer materials. Our technique enables the fabrication of advanced 2D structures with arbitrary boundaries, dimensions as small as 30 nm, and fully customizable energy landscapes. Our optical characterizations further showcase the potential for implementing tailored optoelectronics in these engineered 2D crystals.

## Graphical abstract

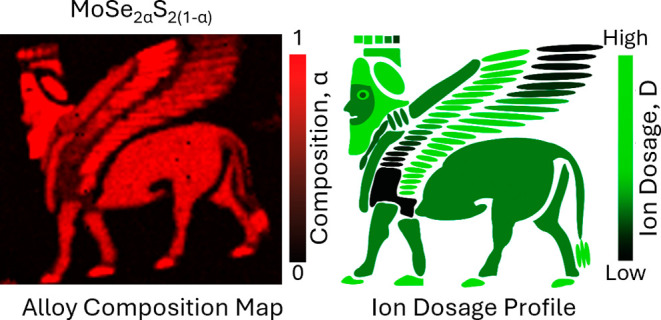

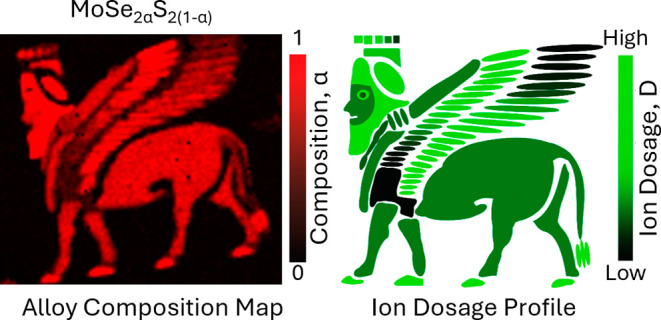

Advancements in optoelectronics rely on the ability to precisely control both geometric parameters and the material composition in heterostructured platforms. A prime example is double-heterostructure laser diodes,^1,2^ which employ heteroepitaxial encapsulation of a low-band-gap active material, typically Al_α_Ga_1−α_As, between two wider-band-gap layers of Al_β_Ga_1−β_As (where β > α). The composition α determines the laser’s emission wavelength, while the sandwiched geometry ensures efficient carrier recombination and optical confinement within the active region. Further engineering of the interplay between the composition and geometry enabled the development of quantum-well,^3,4^ quantum-wire,^5^ and quantum-dot^6^ lasers, revolutionizing optoelectronics. This trajectory of success motivates efforts to achieve a comparable level of control in emerging optoelectronic materials.

Layered transition-metal dichalcogenides (TMDs) and their heterostructures are the newest class of optoelectronic materials, in which their atomically thin nature yields novel quantum effects and unique device functionalities previously deemed impossible in bulk materials. Distinct properties such as strong excitonic effects,^7–9^ fast carrier dynamics,^10,11^ direct optical band gap,^12^ composition diversity (MX_2_, where M = Mo and W and X = S, Se, and Te),^13–15^ and the possibility of cointegration on virtually any substrates give TMD heterostructures a competitive advantage in optoelectronic applications and quantum science. However, to fully leverage this plethora of unique properties for practical applications, simultaneous control over the geometric aspects and compositional properties of TMD heterostructures, particularly on the nanoscale, is mandatory. This, however, is currently an outstanding challenge in the two-dimensional (2D) material community. The widely adopted technique for preparing 2D heterostructures is mechanical exfoliation and manual assembly,^16–18^ which makes systematic control of the geometric aspects and energy structure difficult. To gain better control over material properties, various methods have been explored for the direct growth of TMD heterostructures.^13,16,19–23^ However, challenges such as the random nucleation of heterojunctions,^14,20^ the restricting role of crystal symmetries on obtainable geometries,^24^ and the lack of an engineerable tuning knob limit the capacity of direct-growth approaches in the controlled formation of 2D heterostructures with the desired attributes. Further innovations such as modified edge epitaxy,^25^ laser templating,^26^ and fast and automated control of reaction agents^27^ have introduced additional control. However, these developments target only specific material aspects, lacking a comprehensive solution for controlling the entire design parameters important in optoelectronics of 2D heterostructures. Thus, developing a holistic and scalable approach capable of simultaneously programming both geometric parameters and electronic structures of 2D heterostructures with nanoscale precision is essential for further advancements in 2D materials research.

Here, we introduce a synthetic pathway toward flexible engineering of in-plane properties of 2D TMDs, offering full control over geometric parameters (shape, dimensions, and locations) and, independently, on the material composition and band-gap energy without any inherent limitations imposed by geometry on composition or vice versa. Our technique is based on the defect-mediated transformation of a binary TMD monolayer, such as MoSe_2_, into a ternary MoS_2α_Se_2(1−α)_ alloy with a composition α that is tunable with the level of defects deliberately introduced into the host MoSe_2_ film. We implement this strategy by introducing defects via focused-ion-beam (FIB) irradiation of monolayer MoSe_2_ (Figure 1a, middle) followed by annealing in a sulfur-rich ambient (Figure 1a, bottom). We show that the sulfurization step ensures the exclusive introduction of sulfur into only ion-irradiated regions while preserving the composition of MoSe_2_ films in pristine parts, enabling the localized modulation of material properties within the 2D plane. The use of FIB offers full and independent control over the geometry and energy landscape in two ways. First, by merely modulating the ion dosage (*D*, $\frac{\#\;\mathrm{ions}}{\mathrm{area}}$ ), we exercise complete control over the material composition and band-gap energy of MoS_2α_Se_2(1−α)_ monolayers freely from α = 0 (i.e., MoSe_2_, band gap ≈ 1.5 eV) to α = 1 (i.e., MoS_2_, band gap ≈ 1.85 eV), enabling 350 meV band-gap modulation. Second, we leverage the facile beam scanning of the FIB to craft freeform heterostructures characterized by custom shapes and dimensions realized in desired locations. The dual capacity to control material composition and geometric parameters, independently and synergistically, enables the realization of designer optoelectronics, where complex energy landscapes can be synthetically dictated within atomically thin 2D materials.

**Figure 1:**
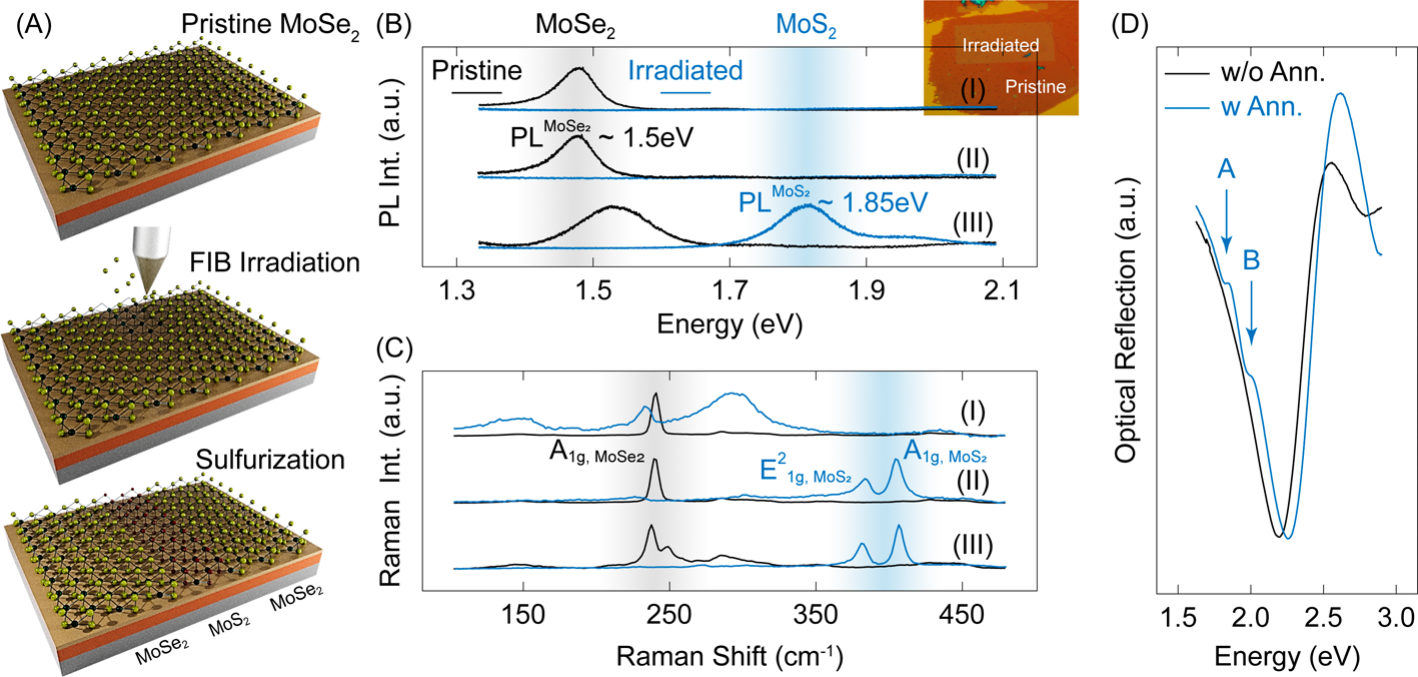
Ion-assisted composition modulation of 2D layers. (a) Schematic representation of the synthesis protocol. (b and c) Normalized PL and Raman spectra of pristine and irradiated (*D* ≈ 2.5 × 10^13^ cm^–2^) MoSe_2_ at three stages: (I) before sulfurization, (II) after sulfurization, and (III) after a sulfurization–annealing sequence. Sulfurization and annealing temperatures are 700 °C (10 min) and 900 °C (5 min), respectively. The inset in panel b shows the optical image of the FIB-irradiated sample. (d) Optical reflectance obtained from two sulfurized samples with and without in situ annealing.

Monolayer MoS_2α_Se_2(1−α)_ alloys can be synthesized via the doping of sulfur into pristine MoSe_2_ films. This approach, however, requires annealing of MoSe_2_ under sulfur gas at elevated temperatures (above ∼800 °C; Figures S1 and S2). However, we demonstrate that the deliberate introduction of defects into a MoSe_2_ host can lower the temperature requirement for sulfur doping and the synthesis of MoS_2α_Se_2(1−α)_ alloys. Accordingly, the localized generation of defects via ion irradiation, followed by a low-temperature sulfurization, enables us to embed MoS_2α_Se_2(1−α)_ alloys within a host MoSe_2_ monolayer and to create lateral heterostructures. The inset of Figure 1b presents an optical image of a MoSe_2_ monolayer that includes an ion-irradiated region (Materials and Methods section). As shown in Figure 1b-I, irradiation induces photoluminescence (PL) quenching, implying the nonradiative recombination of carriers via defect states. The generation of defects is further supported by Raman measurements (Figure 1c-I), wherein the out-of-plane vibration of pristine MoSe_2_ (A_1g,MoSe_2__) at ∼241 cm^–1^ undergoes broadening and a redshift to ∼234 cm^–1^. Additionally, two new spectrally broad features emerge around 145 and 293 cm^–1^. Such spectral changes are attributed to the generation of defects and lattice disorder.^28^ Defects result in a softening of the restoring force acting on Mo–Se bonds, consequently causing a redshift in the A_1g,MoSe_2__ mode. Furthermore, disorder disrupts the selection rules, thereby giving rise to new Raman modes.

After sulfurization at 700 °C, the Raman mode of MoSe_2_ disappears (Figure 1c-II) and the in-plane (E^2^_1g,MoS_2__, 383 cm^–1^) and out-of-plane (A_1g,MoS_2__, 406 cm^–1^) modes of MoS_2_ appear, confirming the formation of Mo–S bonds within the irradiated region. However, the Raman spectrum of the pristine matrix surrounding the irradiated region still exclusively contains the A_1g,MoSe_2__ mode. This outcome proves the selective introduction of sulfur into ion-irradiated regions, which establishes a lateral MoSe_2_–MoS_2_ heterostructure at the interface between the pristine and ion-irradiated regions. However, the PL of the irradiated region still lacks any detectable signal after the sulfurization step (Figure 1b-II), suggesting that the structure of the obtained MoS_2_ is not fully ordered. To address this issue, we added one extra step after the sulfurization; we first purge the sulfur precursor and then subject the sample to in situ annealing at a higher temperature (900 °C, 5 min). In situ annealing yields lattice reconstruction within the irradiated region, while the absence of sulfur gas prevents unintended doping in pristine regions. The 900 °C used in this step is lower than the processing temperatures employed in alternative techniques.^26^ Although follow-up studies are necessary to further reduce the synthesis temperature, well-developed transfer techniques can facilitate the transfer of these engineered layers from the growth substrate to other substrates requiring low-temperature processes.

As illustrated in Figure 1b-III, annealing restores PL emission within the irradiated region, as manifested in the strong light emission at the MoS_2_ band gap of ∼1.85 eV. Furthermore, optical reflectance measurements on the irradiated region reveal the appearance of characteristic A and B excitons of MoS_2_ after the annealing step (Figure 1d). MoS_2_ Raman modes of the irradiated region also exhibit noticeable line-width narrowing following the annealing step (Figure 1c-III). Thus, Raman, PL, and optical reflection measurements confirm the effective recovery of the MoS_2_ lattice following in situ annealing. We acknowledge that the annealing step might induce slight changes in the PL and Raman spectra of pristine regions, perhaps due to residual sulfur doping.

To gain direct insight into structural transformations experienced by MoSe_2_ films during the synthesis process, we conduct scanning transmission electron microscopy (STEM). We transfer MoSe_2_ thin films onto a holey silicon nitride grid (Figure 2a) and irradiate an array of lines onto suspended sections (Figure 2b). We then directly monitor the structural changes in the film via imaging the interface between ion-irradiated and pristine MoSe_2_ before (Figure 2c) and after (Figure 2d) the sulfurization–annealing sequence. Figure 2c indicates the amorphization of MoSe_2_ following FIB irradiation, which explains the previously discussed suppression of PL emission and the redshift and broadening of Raman modes. *After* the sulfurization–annealing sequence (Figure 2d), an ordered and multicrystalline lattice is established within the irradiated region. This lattice reconstruction explains the recovery of PL emission, the appearance of excitonic signatures, and the narrowing of Raman line widths, as we discussed above. We also note that in some scattered locations, as exemplified in Figure 2e, the lattice appears nearly completely reconstructed. However, the reconstruction in most locations reaches the level depicted in Figure 2d. Collectively, we conclude that the localized modulation of the 2D material composition contains three steps (Figure 2f); (i) introduction of Se vacancy via ion irradiation, which also induces lattice deformation, (ii) infiltration of sulfur into Se vacancies during the low-temperature sulfurization, and (iii) lattice reconstruction following the in situ annealing step. We note that the observed amorphous-to-crystalline reconstruction has precedent in other chalcogenide-based compounds, most notably the family of germanium–antimony–telluride and its Se-doped variant (see also the Supporting Information).^29,30^

**Figure 2:**
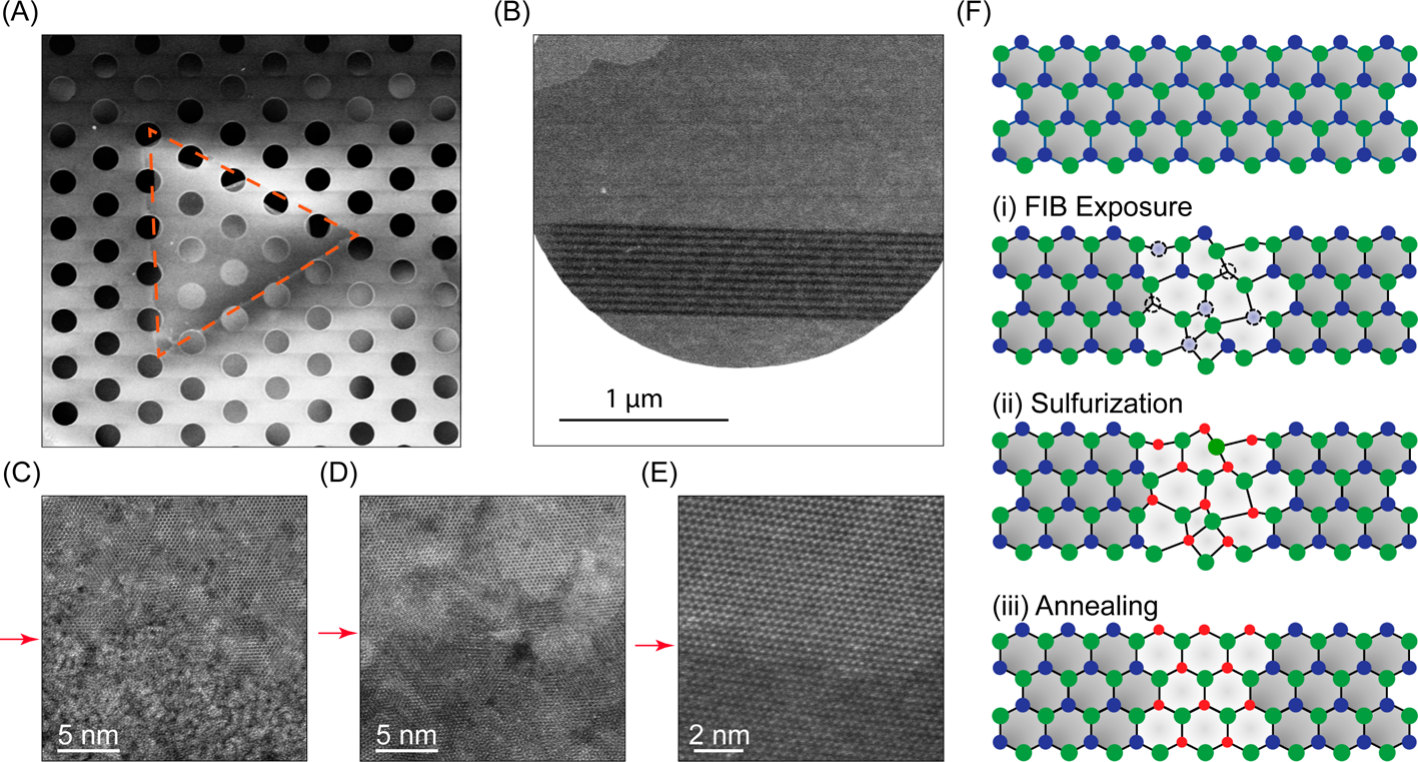
Controlled restoration of the lattice structure. (a) Low-magnification STEM image of a few-layer MoSe_2_ film transferred onto a holey silicon nitride grid. The MoSe_2_ crystal is outlined with a dashed triangle. (b) STEM image of an array of lines (width ≈ 30 nm; pitch ≈ 50 nm) FIB patterned onto a part of the MoSe_2_ crystal that is suspended over a hole. (c and d) High-resolution STEM images at interfaces between FIB-irradiated and pristine MoSe_2_ obtained before and after the sulfurization–annealing sequence, respectively. Arrows point to the interface, and the bottom half of the image is irradiated. (e) Sample interface showing occasional near-complete reconstruction of the lattice. (f) Schematic illustration of structural evolutions at different stages: (i) formation of vacancies (dotted circles) and crystal deformation after FIB irradiation, (ii) selective incorporation of sulfur into the irradiated region after low-temperature sulfurization, and (iii) reconstruction of the lattice after the high-temperature annealing. Green, blue, and red circles represent Mo, Se, and S atoms, respectively.

A distinguishing advantage of our technique is the facile engineering of optoelectronic properties by tuning the material composition. To demonstrate this aspect, we perform the sulfurization–annealing sequence on a sample featuring 10 rectangular regions exposed to ion dosages ranging from *D*_1_ = 0.1 × 10^13^ cm^–2^ to *D*_10_ = 4.4 × 10^13^ cm^–2^. An optical image of the resulting sample is shown in Figure 3a. Raman measurements (Figure 3b) demonstrate that. at high ion dosages, Mo–S vibrations (i.e., A_1g,MoS_2__ and E^2^_1g,MoS_2__) predominate the collected spectra, implying a composition close to that of binary MoS_2_. In contrast, at low ion dosages, the Mo–Se vibration (i.e., A_1g,MoSe_2__) dominates, signaling that the lattice maintains a composition close to the original MoSe_2_. At intermediate dosages, Raman spectra contain partial contributions from both Mo–S and Mo–S bonds, indicating that a ternary MoS_2α_Se_2(1−α)_ alloy is established. Similarly, PL measurements, in Figure 3c, demonstrate that, as the ion dosage increases from *D*_1_ to *D*_10_, the PL peak energy continuously and monotonically blueshifts from the band gap of MoSe_2_ at 1.5 eV toward that of MoS_2_ at ∼1.85 eV. In essence, by merely tuning *D*, we can precisely dictate the composition α and the band-gap energy (*E*_g_) of embedded MoS_2α_Se_2(1−α)_ alloys within the host monolayer film (Figure 3d), achieving compositional control with precision better than ∼10%. Our Raman characterizations (Figure S3) attribute this systematic control to varying levels of irradiation-induced defects in host MoSe_2_ monolayers.

**Figure 3:**
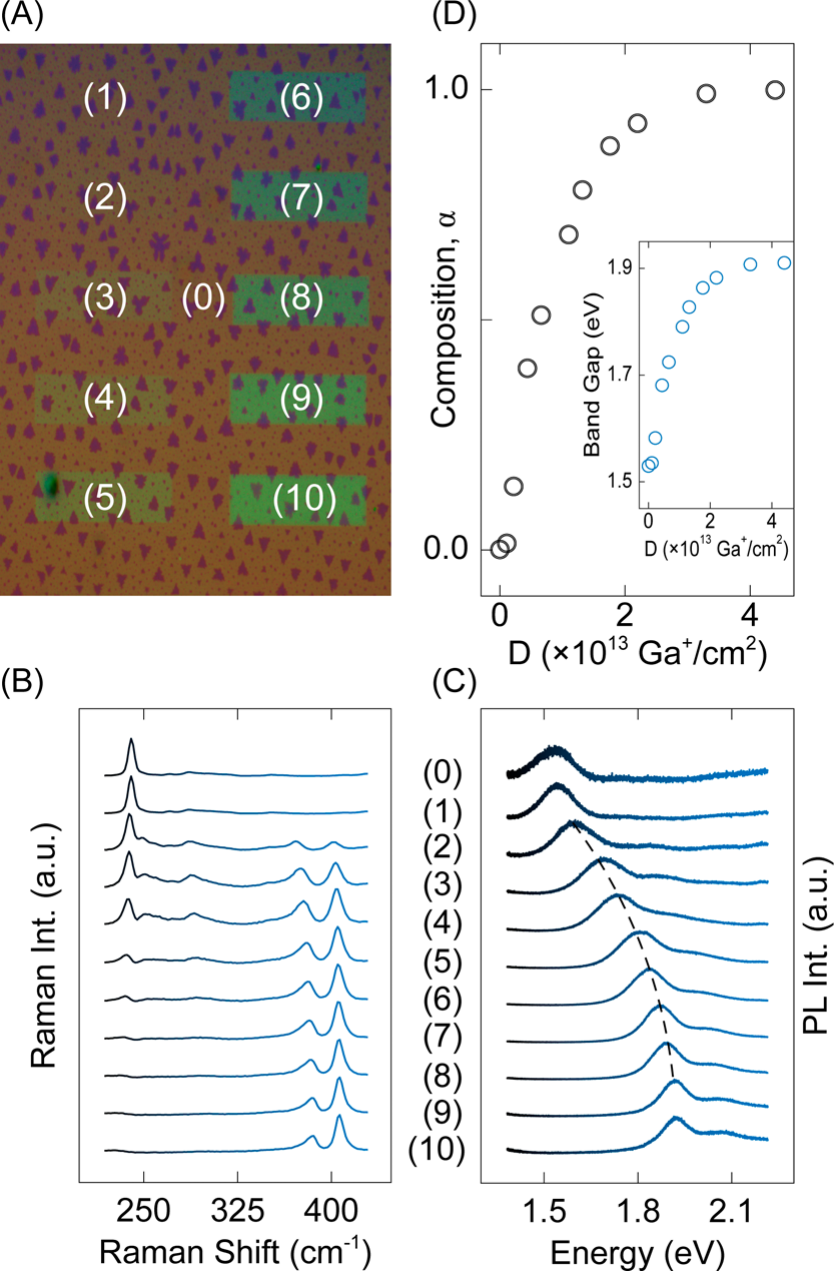
Continuous control of the band-gap energy. (a) Optical image of a representative sample after the sulfurization–annealing sequence. Rectangles indexed as (1)–(10) are irradiated with ion dosages *D*_1_–*D*_10_ = 0.1–4.4 (×10^13^ cm^–2^). Each rectangle is 8 × 22 μm^2^. The region marked as (0) is pristine MoSe_2_. (b and c) Normalized Raman and PL spectra obtained from regions indexed on panel a. For clarity, spectra are displaced vertically. The dashed line on panel c is a guide to the eyes, connecting PL peak positions. (d) Composition α and band-gap energy (inset) of MoS_2α_Se_2(1−α)_ compounds as a function of the ion dosage.

The integration of FIB in our synthesis technique offers an advanced degree of control over the energy landscape within the plane of 2D materials via freeform heterostructuring. We showcase this potential through realizing a complex heterostructure in the form of an ancient Lamassu, the Assyrian deity with the wings of a bird, the body of a lion, and the head of a human, in a literal sense, a “heterostructure” from antiquity. A schematic illustration of the spatial ion-dosage profile and a representative optical micrograph of the printed pattern onto a monolayer MoSe_2_ film are shown in parts a and b of Figure 4, respectively. For realization of the complex heterostructure, the sample subsequently undergoes the sulfurization–annealing sequence. Then, we used optically measured band gaps to determine the composition α of MoS_2α_Se_2(1−α)_ compounds at different parts of the pattern and constructed a composition map in Figure 4c. Three representative Raman spectra obtained from pure MoS_2_, pure MoSe_2_, and mixed Mo–S–Se regions, alongside their atomistic schematics, are presented in Figure 4d. The correlation between the dosage map and the composition map demonstrates the successful synthesis of a complex multicomposition 2D heterostructure. The systematic composition variation in the wings and the large-scale uniformity in the body of the pattern corroborate the high degree of control that our technique offers. Such a piecewise assembly of arbitrarily shaped MoS_2α_Se_2(1−α)_ segments with programmable energy structures is an experimental confirmation of a synthetic pathway toward a designer material platform.

**Figure 4:**
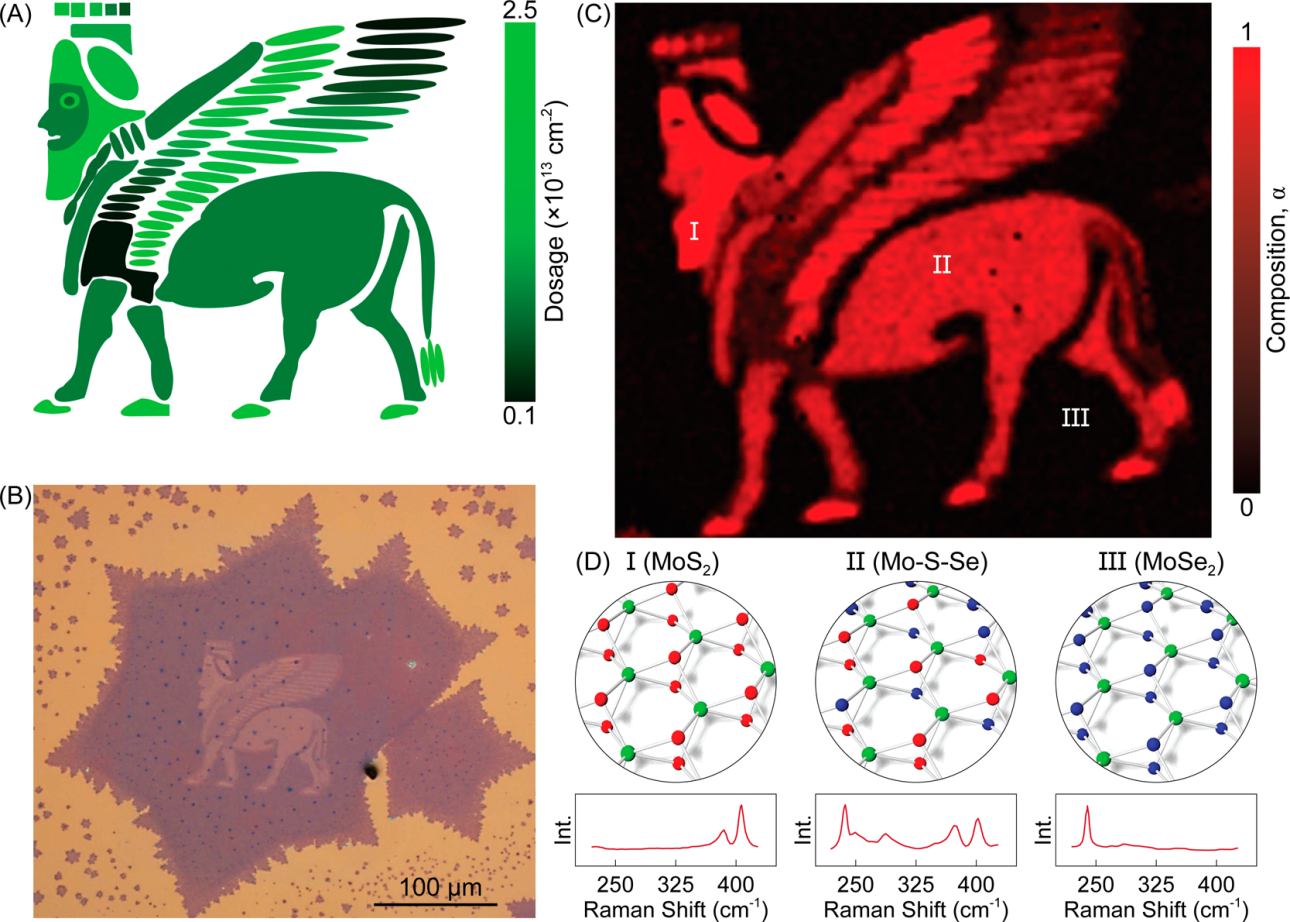
Multicomposition designer materials. (a) Schematic drawing of ancient Lamassu to be FIB-printed onto a MoSe_2_ monolayer. Different ion dosages are represented by different shades of green. (b) Optical micrograph of a representative sample that shows that the pattern is FIB-printed onto the MoSe_2_ monolayer. (c) 2D composition map of the complex in-plane heterostructure established after the sulfurization–annealing sequence. (d) Atomistic schematics (top) and Raman spectra (bottom) of the three points marked on the composition map. Established compounds at points A, B, and C are MoS_2_, Mo–S–Se, and MoSe_2_, respectively. Green, red, and blue spheres represent Mo, S, and Se, respectively.

Here, we highlight the advantages of our ion-assisted technique over alternative methods that generally involve patterned masking and atomic layer substitutions, as originally reported by Mahjouri-Samani et al.,^31^ and further studied by us and others.^32,33^ As shown in Figure S4, using hard masks requires multiple nanofabrication steps: (1) lithographic definition of patterns, (2) material deposition for hard masks, (3) chalcogen replacement, and (4) removal of patterned masks for electrical access to the heterojunction. In a multicomposition heterostructure, these four steps must be repeated for every MoS_2α_Se_2(1−α)_ segment with a different α value. Thus, such a synthesis scheme results in the exponential growth of nanofabrication steps into an unfeasible number when targeting complex heterostructures. In sharp contrast, our approach enables advanced multicomposition heterostructuring (e.g., Figure 4) in a single run without any complex gas delivery, material masking, or sequential nanofabrication steps. Additionally, removal of the hard mask for electrical access to the heterojunctions complicates the device fabrication flow in the patterned-masking approach. Our technique addresses this issue via eliminating hard masks from the synthesis process altogether. Finally, a key advantage of our approach is its fine spatial resolution. The ∼30 nm features demonstrated here are unattainable in the patterned masking technique due to the diffusion of reactive agents underneath the hard masks. We also note that sub-100-nm resolution is readily achievable even on standard oxide substrates, as demonstrated in Figure S5.

We next demonstrate that, despite the multicrystalline nature, our engineered 2D materials yield junctions with optoelectronic properties suitable for photodetection and photovoltaic applications. We analyzed the alignment of electronic bands at a MoS_2_–MoSe_2_ junction via photocurrent mapping experiments on a device shown in Figure 5l. A representative photocurrent map under illumination with λ = 550 nm light and the drain–source voltage (*V*_ds_) of 5 V is shown in Figure 5b. Comparing this photocurrent map with the optical reflection map in Figure 5a reveals that the photocurrent is predominantly confined to the junction area. This implies that photogenerated electron–hole (e–h) pairs separate merely at the vicinity of the junction, indicating the type II band alignment in which electrons and holes move to opposite sides of the MoS_2_–MoSe_2_ junction (Figure 5k). As shown in Figure 5b–e, photocurrent generation requires a threshold voltage of *V*_ds_^thr^ ≈ 2 V, as further highlighted in line cuts across the MoS_2_–MoSe_2_ interface (Figure 5j). Possible mechanisms contributing to formation of the threshold voltage are discussed in the Supporting Information. As illustrated in Figure 5b,g,i, increasing the wavelength of the excitation light from λ = 550 to 620 and 730 nm results in a monotonic reduction of the photocurrent across the junction. This trend stems primarily from a reduced band-to-band light absorption and inefficient e–h pair generation. The systematic dependence of the photocurrent on photon energy further substantiates the creation of well-defined optoelectronic band gaps in the constituting materials at the studied junction.

**Figure 5:**
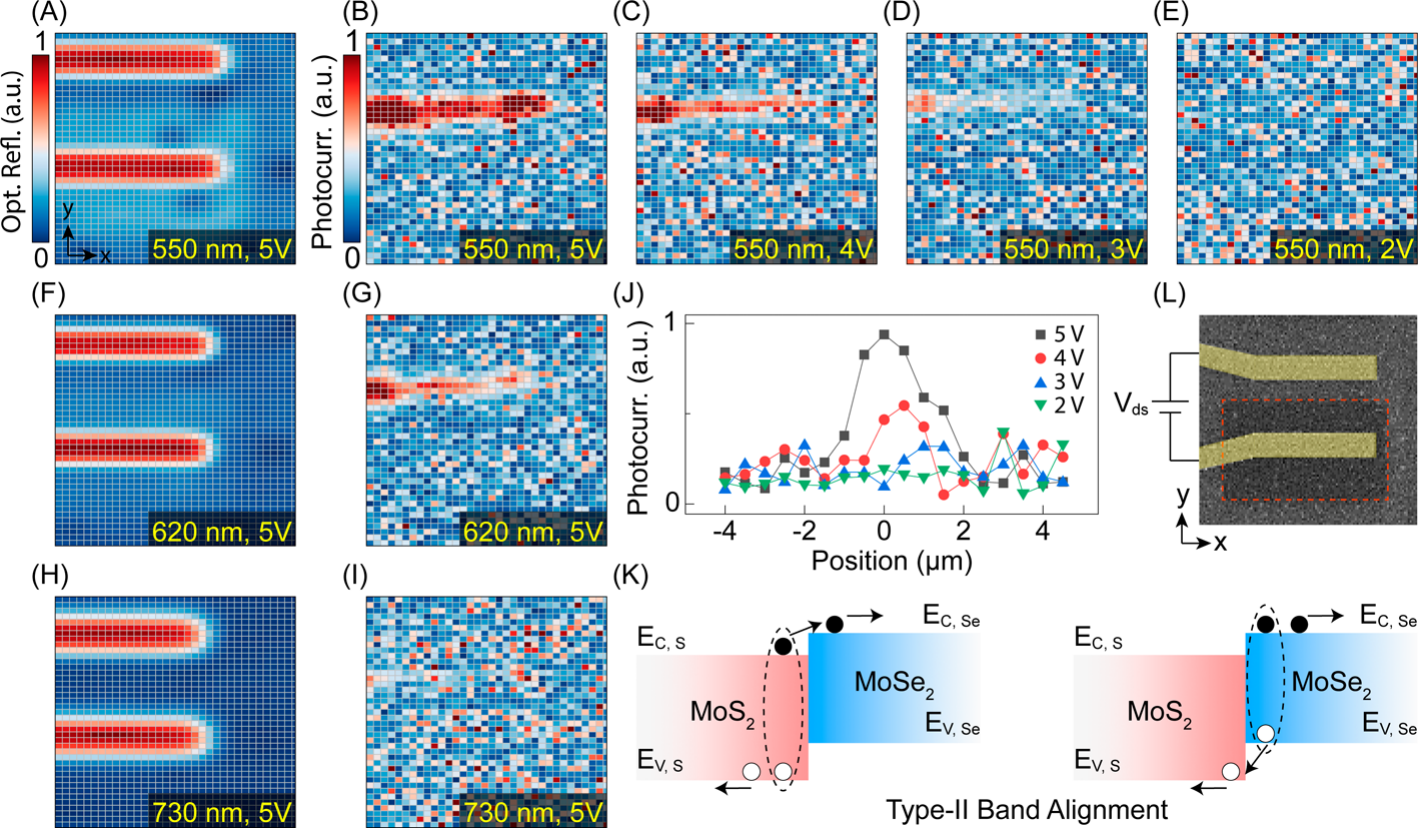
Demonstration of photovoltaic effects. (a) Optical reflection map obtained under illumination with 550 nm light. Drain–source electrodes (Au/Ti) are identified by strong optical reflections. (b–e) Photocurrent maps obtained under fixed wavelength λ = 550 nm and *V*_ds_ = 5, 4, 3, and 2 V, respectively. Optical reflection and photocurrent maps obtained under (f and g) λ = 620 nm and *V*_ds_ = 5 V and (h and i) λ = 730 nm and *V*_ds_ = 5 V. (j) Line cuts of photocurrents across the heterojunction interface at different *V*_ds_ values and a fixed λ = 550 nm. The junction is located at 0 μm. (k) Schematic representation of e–h pair separation across a type II lateral heterojunction. E_c_ and E_v_ represent the conduction- and valence-band edges, respectively. Open and closed circles represent holes and electrons, respectively. (l) Representative SEM image of the measured device. The dashed box outlines the region converted into MoS_2_. Maps are obtained from an area of 17 μm × 20 μm. Photocurrent maps are normalized to the strongest current measured among all (λ and *V*_ds_) combinations. Optical reflection maps are normalized separately for each λ.

Our photocurrent mapping experiments collectively demonstrate that photocurrents are confined to the junction area, exhibit rectifying behavior, and reveal the establishment of well-defined optical band gaps in synthesized heterostructures. These observations indicate that, despite interfacial disorders and the polycrystalline nature, the key characteristics of type II band alignments in lateral junctions are preserved in heterostructures produced by our ion-assisted approach. Thus, while the full crystallinity of lateral junctions is preferred, it is not a requirement for impacting the photovoltaic applications of 2D materials. For instance, in the mainstream silicon (Si) photovoltaic technology, the optimal efficiency of single-crystalline Si is compromised for the benefit of lower-cost production with *polycrystalline* Si. Similarly, our ion-assisted technique can potentially yield polycrystalline 2D heterostructures in which the lack of full junction crystallinity is balanced against the extra advantages of finely tunable band-gap energy as well as designable geometric aspects of 2D junctions. However, the polycrystallinity might impose performance trade-offs on charge transport, carrier recombination, and overall device response, which require follow-up studies.

In conclusion, our introduced ion-assisted synthetic approach offers flexible control over the properties of 2D layers beyond their intrinsic forms. The ability to locally modulate the material composition within complex geometric profiles grants material designers full access to the entire design parameters, enabling the conception of more advanced device structures. On the one hand, our synthesis technique is scalable to large areas, only limited by the size of the host 2D materials (approximately 100 μm in our samples; Figure 4). On the other hand, the spatial resolution of FIB microscopes allows for nanometer-scale precision, as demonstrated by our ability to achieve dimensions as small as tens of nanometers in suspended films (Figure 2b). Replacing Ga^+^ with He^+^ ions can further push boundaries to dimensions approaching the ion-beam size of ∼0.5 nm.^34^ Capitalizing on this resolution, our ion-assisted method offers a potentially transformative pathway toward unlocking novel quantum-confinement effects that emerge at length scales approaching the exciton Bohr radius of 2D TMDs (a few nanometers). While ion–substrate interactions might enlarge the irradiated area,^35^ the bending of electronic bands at the lateral heterojunctions can tighten spatial confinement to dimensions smaller than metallurgical dimensions. This further underscores another facet of our approach, that is, full control over tuning of the electronic structure via merely adjusting the ion dosage. Such distinct features offer a promising pathway toward the scalable realization of quantum dots and quantum wells in a deterministic and adaptable fashion with tunable optoelectronic properties via the facile design of geometry and energy landscape. However, fully realizing such heterostructure platforms for quantum applications requires improved control over the crystallinity and interfacial order. Our work further highlights the broad utility of the FIB technique for nanofabrication and material processing^36^ and suggests its potential to advance research in areas such as spintronics and magnonics,^37^ where the excitation of spin waves with nanometer-scale wavelengths is critical.

## Supplementary Material



## Notes

Materials and methods, schematic representation of the sulfurization setup, direct sulfurization at high temperatures, Raman analysis of controlled defect introduction, comparison of the FIB approach with conventional lithography-based heterostructuring techniques, discussion on the amorphous-to-crystalline reconstruction, discussion on the origin of the voltage threshold in photocurrent mapping experiments, and discussion on the band-gap modulation with the alloy composition (PDF)
