# Supplementary material for: Ion-Assisted Nanoscale Material Engineering in Atomic Layers
Source: Nano Lett. 2025 Jun 13;25(25):10123–30. doi: 10.1021/acs.nanolett.5c02040 (PMC12203579; doi:10.1021/acs.nanolett.5c02040)
Supplement: Supplementary file 1 [file nl5c02040_si_001.pdf]

Supporting Information

Ion-Assisted Nanoscale Material Engineering in Atomic Layers

Hossein Taghinejad, [1,2,3,*] Mohammad Taghinejad, [2,4,5] Sajjad Abdollahramezani, [2,4] Qitong Li, [5] Eric V. Woods, [6,7] Mengkun Tian, [6] Ali A. Eftekhar, [2] Yuanqi Lyu, [1] Xiang Zhang, [8] Pulickel M. Ajayan, [8] Wenshan Cai, [2] Mark L. Brongersma, [5] James G. Analytis, [1,9,*] Ali Adibi [2,*]

1. Department of Physics, University of California, Berkeley, CA 94720, USA.
2. School of Electrical and Computer Engineering, Georgia Institute of Technology, Atlanta, GA 30332, USA.
3. Kavli Energy NanoSciences Institute, University of California, Berkeley, CA 94720, USA.
4. School of Materials Science and Engineering, Stanford University, Stanford, CA 94305, USA.
5. Geballe Laboratory for Advanced Materials, Stanford University, Stanford, CA 94305, USA.
6. Institute of Electronics and Nanotechnology, Georgia Institute of Technology, Atlanta, GA 30332, USA.
7. Max Planck Institute for Iron Research, Max-Planck-Straße1, Düsseldorf 40237, Germany.
8. School of Materials Science and Nanoengineering, Rice University, Houston, TX 77005, USA.
9. Materials Sciences Division, Lawrence Berkeley National Laboratory, Berkeley, CA 94720, USA.

*Corresponding Authors:
h.taghinejad@berkeley.edu
analytis@berkeley.edu
ali.adibi@ece.gatech.edu

Materials and Methods

Focused-ion-beam irradiation. Ion irradiation experiments were conducted in a dual-column Gallium ion (Ga^+) FIB/SEM microscope (FEI Nova NanoLab 200) at a fixed acceleration voltage of 5 kV and varying ion-beam currents (I) of 5 pA, 20 pA, or 80 pA. To ensure uniform exposure in large-area irradiations, we intentionally increase the beam area (A) by introducing defocusing. We also deliver an intended ion dosage in multiple passes (N_p). Accordingly, the ion dosage is calculated as $D = \frac{I t_d N_p}{qA}$, where t_d and q are the dwell time and the Ga^+ charge, respectively. [1] Sample navigations and imaging were conducted using the electron beam (i.e., the SEM mode). For experiments presented in Figure 4, we rely on the pattern generator of the FIB system to scan the ion beam over an arbitrarily complex spatial profile defined within an input bitmap file. Guided by the bitmap file, the pattern generator sends signals to the electrostatic deflection plates inside the FIB column for vector-scanning the ion beam over $MoSe_2$ monolayers. Each pixel in the bitmap file contains a combination of red, green, and blue (R, G, B) integer values, each varying between 0 and 255. The R content is not currently used by our pattern generator, and we keep it constant at 0. The G content determines the dwell time t_d, which in turn defines the delivered ion dosage (for a given I and N_p) and eventually the composition of the $MoS_{2\alpha}Se_{2(1-\alpha)}$ lattice at each pixel. t_d is 0.1 μSec at G = 0, and it linearly increases with increasing the G value. Finally, the B content controls the beam blanker, and for any nonzero value the beam is unblanked. Accordingly, we use the (0, G, 255) combination for pixels residing within our desired pattern and (0, 0, 0) everywhere else.

Sulfurization and annealing processes. For the sulfurization step, we load sulfur powder into a quartz crucible and place it next to SiO_2/Si substrates covered with CVD-grown $MoSe_2$ monolayers. Then we warm up the chamber from room temperature to a targeted sulfurization temperature and hold it there for approximately 10mins. For annealing steps, we first flush the reaction chamber by steadily flowing Ar gas into the chamber to deplete sulfur gas. Then, the temperature is elevated to ~900 ^{o}C for annealing the sample for ~5mins. Eventually, samples are cooled to room temperature.

Optical characterization. PL and Raman measurements were performed using a Renishaw inVia confocal microscope with a laser excitation wavelength of 532 nm focused down to a ~1 μm diameter spot using a 100X objective lens. The laser power is kept below 100 μW to mitigate laser heating. The composition α of $MoS_{2\alpha}Se_{2(1-\alpha)}$ compounds (shown in Figure 3d) is extracted from optical bandgaps (E_g) in PL measurements. Assuming a linear modulation with negligible band-bowing, [2] we extract α from $E_{g,\alpha} = (\alpha)\, E_{g,MoS2} + (1-\alpha)\, E_{g,MoSe2}$. Optical reflection measurements are performed at a normal incident angle using a 50X objective lens with a numerical aperture of ~ 0.5. A tungsten halogen lamp is used as the source, and a Craic QDI 202 micro-spectrophotometer mounted on a Leica DM 4000M microscope serves as the detector.

Photocurrent mapping. Photocurrent mappings are carried out in a custom-built optoelectronic setup. A supercontinuum laser with two acousto-optic tunable filters (Fianium) is used to tune the wavelength of monochromatic light used for photoexcitation of 2D heterostructures. A 5X long-working-distance objective lens (Mitutoyo M Plan APO, 0.14 NA) focuses the light onto the sample. To image the sample, two 50:50 beam splitters, a

halogen lamp with a diffuser, and a CCD imaging camera with tube lenses are placed before the objective. Samples are wire-bonded to a chip carrier and then mounted on a three-axis piezo stage with a rotating platform to accurately focus the beam spot at the center of the devices. A source meter (Keithley 2612) is used to extract the photocurrent from the sample biased at different V_{ds} voltages.

STEM imaging. The STEM imaging is performed in an 80 kV aberration-corrected Hitachi HD2700 microscope operated in the high-angle annular dark field (HAADF) mode. Beam current is kept below 30 pA to minimize the ionization damage. To enable high temperature processes, $MoSe_2$ films are transferred onto holey grids made of SiN.

Device fabrication. Standard electron-beam lithography (EBL) using Poly(methyl methacrylate) (PMMA) is used for definition of electrical contacts. Then, Au/Ti (60 nm/10 nm) metal contacts are deposited via e-beam evaporation, followed by a liftoff in acetone for resolving contacts. To ensure Ohmic contacts, devices are annealed at 200 ^{o}C under the flow of H_2 gas diluted in Ar.

Amorphous-to-Crystalline Reconstruction:

The annealing driven amorphous-to-crystalline reconstruction observed in our studied Mo-S-Se ternary system is consistent with previous findings in various chalcogenide-based compounds. Among these compounds, the most well-known is the family of germanium-antimony-telluride, including its Se-doped variant. [3, 4] The crystallization process in an amorphous lattice involves two main stages: (i) the nucleation of small crystallites and (ii) the subsequent growth and enlargement of these nuclei. Nucleation occurs thermodynamically below the material's melting point, where the free energy difference (between amorphous and crystalline phases) favors the crystalline phase. As the temperature drops from the melting point, the nucleation rate increases and reaches its maximum near the glass-transition temperature. Considering the nucleation requirement alone, one might then expect the amorphous-to-crystalline reconstruction to occur even during a low-temperature sulfurization step. However, the high nucleation rate at low temperatures does not lead to significant crystal growth due to the slow atomic mobility. In essence, although nucleation does take place, the slow expansion of crystals around nucleation centers impedes substantial lattice reconstruction during the low-temperature sulfurization step. However, our introduced in-situ annealing step at a higher temperature offers the required balance between the two criteria, providing a sufficient thermodynamic driving force for the nucleation as well as the fast atomic mobility for the rapid crystal growth during the annealing period.

Controlled Introduction of Defects

Figure S3 demonstrates our ability to employ the ion dosage D as a tuning knob for modulating the defect level in monolayer $MoSe_2$ crystals through FIB irradiation. We study this aspect via analyzing characteristic changes in the $A_{1g,\ MoSe2}$ Raman mode of $MoSe_2$ films subjected to varying dosages of Ga^+ ions. Increasing the ion dosage induces several systematic changes to the $A_{1g,\ MoSe2}$ Raman mode: (1) the reduction of the integrated intensity (panel (D)), (2) the redshift of the peak position (panel (E)), and (3) the broadening of the Raman linewidth (panel (F)). These characteristic modifications unanimously indicate that increasing the ion dosage increases the level of defects and disorder in irradiated $MoSe_2$ films. The linear trends in response to varying D indicate the binary collision regime, which is typical of low-dosage ion irradiation. Ion irradiation leads to the sputtering of atoms from the $MoSe_2$ lattice. However, details of ion interactions with 2D materials are very subtle and the SiO_2/Si substrate underneath the 2D film adds further complexities. Nonetheless, the nature of the $A_{1g,\ MoSe2}$ vibration can provide insight into the sputtering of atoms from $MoSe_2$ following the irradiation. As shown in the inset of panel (A), the $A_{1g,\ MoSe2}$ mode stems from the out-of-plane breathing vibration of Se atoms relative to the metal plane, making the A_{1g} Raman mode very sensitive to details of the Se sublattice. Thus, the consistent drop of the integrated intensity of the $A_{1g,\ MoSe2}$ mode primarily implies the creation of Se-vacancies with a density that is linearly controllable with the ion dosage. We believe that these Se-vacancies serve as entry points which facilitate the subsequent sulfur intake during our low-temperature sulfurization process. As such, the D-dependent loss of Se following the FIB irradiation forms the foundation of the systematic control we have over the composition of

$MoS_{2\alpha}Se_{2(1-\alpha)}$ heterostructures as we have demonstrated in Fig. 3 and Fig. 4 of the main manuscript. In other words, a higher density of Se-vacancies, introduced at higher ion dosages yields $MoS_{2\alpha}Se_{2(1-\alpha)}$ compounds with larger α values (Fig. 3, main text). We note that the ion irradiation of monolayer TMDs is more likely to generate chalcogen vacancies than metal vacancies consistent with the existing knowledge in literature. [5] As shown in detailed calculations performed on monolayer TMDs, molecular dynamics simulations combined with density functional theory and Monte Carlo calculations suggest two primary reasons for such a sputtering selectivity. First, the projectile ion (i.e., Ga^{+}) needs to transfer a minimum kinetic energy, known as the displacement threshold (T_d), to atoms in target materials to knock them out of the crystal lattice. This displacement threshold is significantly smaller for chalcogen atoms than metal atoms in a monolayer TMD. Second, the cross-section to produce metal vacancies is significantly smaller than that for generating chalcogen vacancies. Therefore, although non-zero, the combination of the smaller cross-section and the larger displacement threshold makes it harder to generate metal vacancies as compared to chalcogen vacancies. We also note that previous experiments conducted on various TMDs conclude that the creation of defects and vacancies are more challenging in substrate supported $MoSe_2$ monolayers than in suspended films. [35] Within the binary collision approximation, this difference arises because the substrate impedes the forward motion of Se and Mo atoms following the momentum transfer from incoming Ga^{+} ions. Thus, it becomes harder for Se and Mo atoms to exit the lattice and create vacancies on substrate-supported films. More importantly, this substrate effect disproportionately influences the generation of Mo vacancies compared to

Se vacancies. Considering the distance of elements from the substrate in the tri-layer Se – Mo – Se configuration of $MoSe_2$, Se atoms in the top Se-layer experience minimum influence from the substrate, unlike the Mo-layer and the bottom Se-layer. Such a disproportionate substrate influence further contributes to the preferential creation of Se-vacancy over the Mo-vacancy. Additionally, due to the trigonal prismatic coordination of $MoSe_2$ monolayers, Mo atoms are connected to six Se atoms, while Se atoms form bounds with only three Mo atoms. This structural arrangement also favors the creation of Se vacancies compared to the Mo vacancies.

Note 1: that according to the PL measurements (Figure 3, main text), a full conversion is achieved at an ion dosage of ~1.5×1013. At this ion dosage, the Raman intensity (Figure S3) drops by less than ~50%, suggesting that more than half of the Mo-Se bonds are still present in the sample. This comparison confirms that full conversion does not require sputtering entire Se atoms out of the lattice.

Note 2: The precision in tuning the alloy composition α is governed by both the accuracy of FIB-induced defect densities and the complex dynamics of sulfur incorporation during the post-irradiation process. As shown in Figure S3, Raman characteristic spectral features (e.g., intensity, FWHM) scale linearly with ion dose, suggesting that FIB irradiation allows highly precise and potentially ultrafine control over initial defect density. However, the final α value (after sulfurization) reflects additional non-linear effects such as defect–defect interactions, secondary vacancy formation, and local variations in sulfur diffusion and reactivity. These factors introduce precision limits, with achievable $\Delta\alpha \approx 10\%$ at low ion doses and $\Delta\alpha \approx 5\%$ at higher α values, as demonstrated in Figure 3, main text.

Voltage Threshold in Photocurrent Mapping Experiments:

Photocurrent generation in type-II band alignments within bulk semiconductors does not, generally, necessitate an external bias. Upon photoexcitation, free electron-hole pairs form and then separate at the type-II junction, generating photocurrent without the need for an external bias. In contrast, in 2D materials, photoexcited carriers exist as excitons—tightly bound electron-hole pairs. Therefore, generating photocurrent in 2D type-II heterostructures requires breaking these exciton bonds to free the electrons and holes, a process facilitated by an applied bias. This distinction is crucial for understanding the role of the bias field in our measurements. For instance, assuming a junction length of 10 nm (the distance over which band bending occurs at the MoS_2-$MoSe_2$ junction), an applied 2V bias creates an electric field of $E = 2V/10nm = 2\times10^6$ ($V.cm^{-1}$). This field is strong enough to overcome the exciton binding energy in TMDs. However, precise values cannot be provided because estimating the effective junction length is difficult. Nonetheless, the order-of-magnitude estimates are reasonably within the range of exciton binding energies reported in the literature. Therefore, we believe the presence of a threshold voltage for observing photocurrents in our devices primarily reflects an inherent property of 2D materials, the excitonic behavior. It is important to note that the threshold voltage required to observe photocurrent in our devices is relatively low. The distance between the source and drain electrodes is approximately 8 μm, and the threshold voltage is below 2V. This means an external field of only 0.25 V/μm is sufficient to generate photocurrent. Therefore, we believe such a small threshold bias will not impose any restrictions on the practical applications of these 2D heterojunctions. Furthermore, a fraction of the applied voltage drops across the

series resistances on both sides of the junction, as shown in Figure S7. Thus, the measured ~2V threshold voltage does not entirely drop across the junction area.

Additionally, we the presence of interfacial states, particularly given the polycrystalline nature of the junction, and trap-assisted recombination could raise the effective potential needed for the observation of photocurrent. These effects are not easily deconvolved without further measurements such as temperature-dependent photocurrent experiments to distinguish between thermally activated transport and field-induced exciton dissociation.

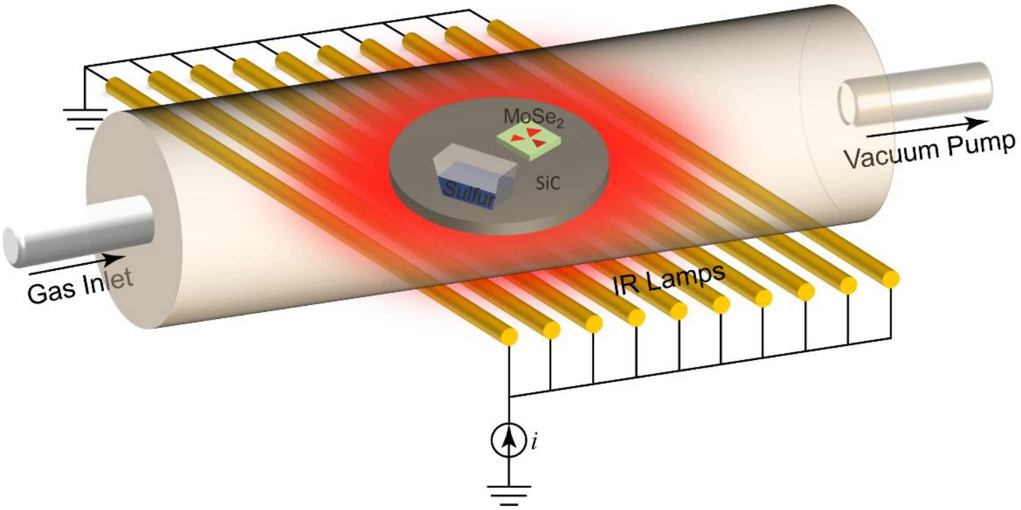

Figure S1. Schematic Illustration of the Sulfurization Furnace. A SiO_2 (90 nm)/Si substrate covered by monolayer $MoSe_2$ films and a crucible containing sulfur powders are placed next to each other on a silicon carbide (SiC) stage. The SiC stage absorbs radiation from an array of Infrared (IR) lamps integrated underneath the furnace, leading to the heating of the sample and sulfur crucible. By changing the current (i) driven into the IR lamps we control the radiation power and accordingly the sample temperature. A closed-loop feedback system reads the sample temperature (through a thermocouple placed near the sample) and accordingly adjusts the current driven into the IR lamps towards achieving a targeted temperature.

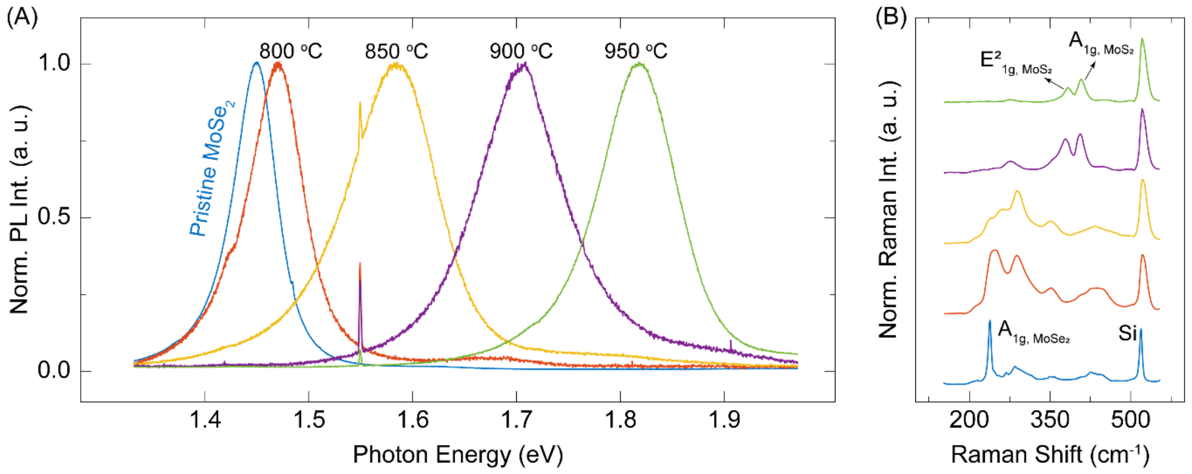

Figure S2. High-Temperature Sulfurization of Pristine $MoSe_2$ Monolayers (No FIB Irradiation). (A) Photoluminescence (PL) and (B) Raman spectra of $MoSe_2$ monolayers sulfurized at various temperatures for 5-10 mins. Noticeable incorporation of sulfur in the host $MoSe_2$ monolayer, manifested in the blueshift of the PL peak position, is observable when the sulfurization is conducted above 800 °C. The sharp features around 1.55 eV are artifacts of the employed grating in the spectrometer. In panel (B), increasing the sulfurization temperature leads to the gradual disappearance of the $A_{1g,\ MoSe2}$ Raman mode of the host $MoSe_2$ film and the emergence of the $A_{1g,\ MoS2}$ and $E^2_{1g,\ MoS2}$ modes of MoS_2. The Raman mode of silicon at ~520 cm^{-1} is displayed as the reference.

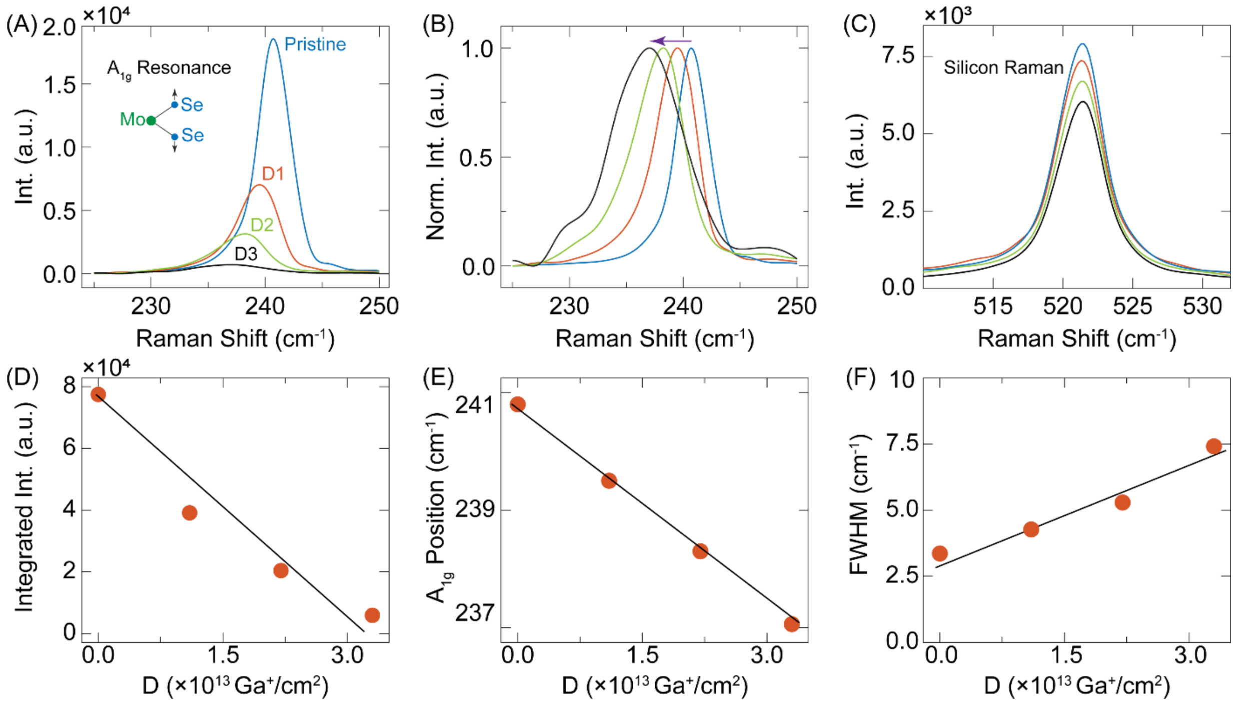

Figure S3. Raman Analysis of The Defect Level Controlled by The Ion Dosage. (A) The $A_{1g,\ MoSe2}$ Raman mode of $MoSe_2$ monolayers subjected to different ion dosages of D_1, D_2, and D_3 = 1.1, 2.2, and 3.3×10^{13} cm^{-2}, respectively. The Raman mode of pristine $MoSe_2$ (centered at 241 cm^{-1}) is shown for comparison. The inset schematically illustrates the out-of-plane $A_{1g,\ MoSe2}$ vibration. (B) Normalized Raman spectra, highlighting redshift and broadening of the $A_{1g,\ MoSe2}$ linewidth as the ion dosage increases. The arrow points to the direction of increasing ion dosage. (C) The Raman spectra of silicon substrate centered at ~ 521 cm^{-1} is measured concurrently with $A_{1g,\ MoSe2}$ for calibration purposes. Identical color codes are used in panels (A)-(C). (D-F) Integrated intensity (i.e., the area under the spectrum), peak position, and the full width at half maximum (FWHM), respectively, of the $A_{1g,\ MoSe2}$ at different ion dosages.

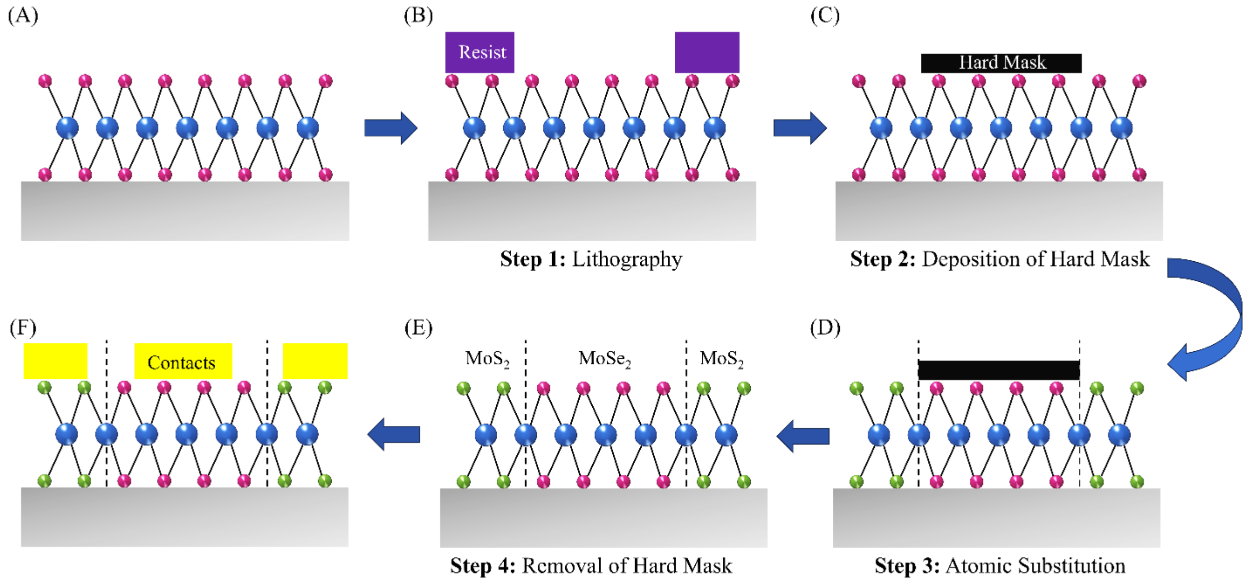

Figure S4. Schematic Illustration of Nanofabrication Flow in Patterned Masking and Atomic Substitution. (A) Starting material: monolayer TMD films, in this example, $MoSe_2$. (B) Step 1: Lithographic definition of the desired pattern in a sacrificial resist. Electron beam and photolithography can be employed. (C) Step 2: Deposition of the physical hard mask into the lithographically defined pattern. The hard mask must be inert during the atomic substitution reaction, withstand high temperatures, and form tight contacts with the underlying 2D material to block diffusion of reactive agents. Silicon dioxide (SiO_2) is commonly used for this purpose. (D) Step 3: Atomic substitution process. This example illustrates the substitution of Se atoms by S atoms, converting $MoSe_2$ into MoS_2. The hard mask restricts atomic substitution to only the uncovered regions of the starting monolayer TMD, resulting in the formation of a lateral heterostructure. Techniques such as annealing and remote plasma treatment in the presence of chalcogen-containing agents (e.g., H_2S or pure S gas) are popular methods for this step. (E) Step 4: Removal of the hard mask to access the junction area for potential device fabrication as shown in (F). We note that each of the four main steps shown in this process flow consists of multiple sub-steps, which we have not shown for simplicity. We also note that the atomic substitution can be stopped at an intermediate stage and convert the starting $MoSe_2$ compound to a $MoS_{2\alpha}Se_{2(1-\alpha)}$ compound. for the synthesis of a multi-composition heterostructure (i.e., a heterostructure containing different α values), these four steps should be repeated for each α value. Thus, as the number of α values increases, the number of required nanofabrication steps will accordingly increase exponentially. Our FISH technique can generate any number of compositions in only one single cycle, without any complexity. We achieve this flexibility by delivering a nonuniform ion dosage prior to the sulfurization step, as shown in Figure 3 and 4 in the main text.

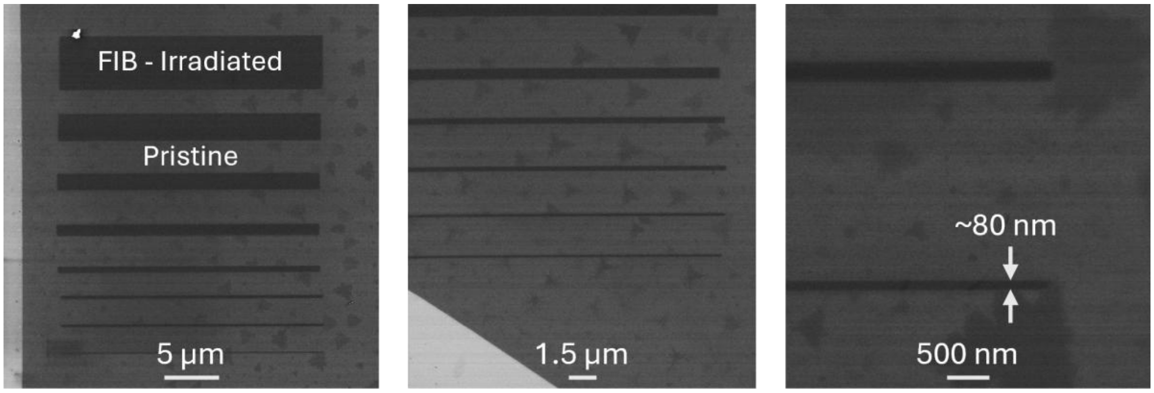

Figure S5: SEM Images of FIB-Irradiated Lines on Monolayer $MoSe_2$ Supported by SiO_2/Si. Irradiated regions, converted to MoS_2, appear darker due to material contrast. Feature sizes as small as tens of nanometers are demonstrated, showing that sub-100 nm resolution is readily achievable even on standard oxide substrates.

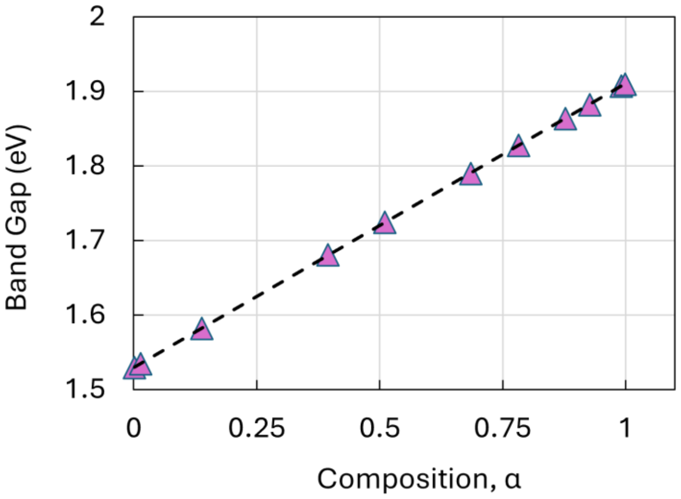

Figure S6: Band Gap Modulation in $MoS_{2α}Se_{2(1-α)}$ Alloys. The band gap energy (E_g) varies linearly with alloy composition α, following $E_{g,\,α} = (α)\,E_{g,\,MoS2} + (1-α)\,E_{g,\,MoSe2}$. The measured values $E_{g,\,MoSe2}$ ≈ 1.53 eV and $E_{g,\,MoS2}$ ≈ 1.9 eV for $MoSe_2$ and MoS_2 binary compositions, respectively, are consistent with literature reports. [6] The linear trend confirms negligible band gap bowing, in agreement with prior theoretical [2, 7] and experimental [6] studies on Se-Mo–S ternary alloys.

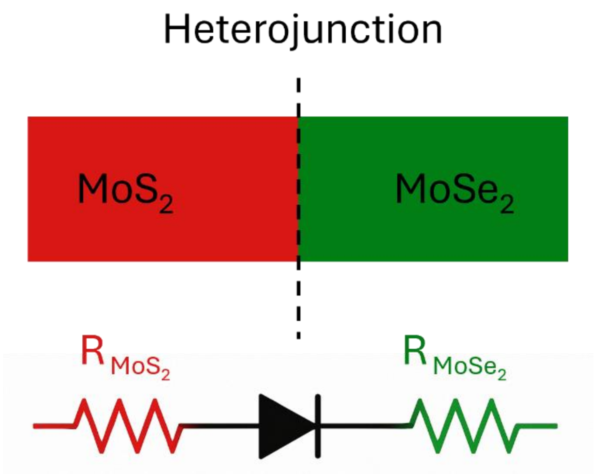

Figure S7: Simplified Electronic Circuit Representing the MoS_2-$MoSe_2$ Photodetector. Schematic representation of the MoS_2–$MoSe_2$ photodetector modeled as two series resistors and a rectifying junction (diode). The applied voltage is divided between the resistive segments and the active heterojunction.

References:

[1] Thiruraman, J. P.; Masih Das, P.; Drndić, M. Irradiation of Transition Metal Dichalcogenides Using a Focused Ion Beam: Controlled Single-Atom Defect Creation. *Adv Funct Mater* **2019**, *29* (52), 1904668.

[2] Kang, J.; Tongay, S.; Li, J.; Wu, J. Monolayer Semiconducting Transition Metal Dichalcogenide Alloys: Stability and Band Bowing. *J Appl Phys* **2013**, *113* (14), 143703.

[3] Zhang, Y.; Chou, J. B.; Li, J.; Li, H.; Du, Q.; Yadav, A.; Zhou, S.; Shalaginov, M. Y.; Fang, Z.; Zhong, H.; Roberts, C.; Robinson, P.; Bohlin, B.; Ríos, C.; Lin, H.; Kang, M.; Gu, T.; Warner, J.; Liberman, V.; Richardson, K.; Hu, J. Broadband Transparent Optical Phase Change Materials for High-Performance Nonvolatile Photonics. *Nat Commun* **2019**, *10* (1), 4279.

[4] Wuttig, M.; Yamada, N. Phase-Change Materials for Rewriteable Data Storage. *Nat. Mater.* **2007**, 6, 824–832.

[5] Kretschmer, S.; Maslov, M.; Ghaderzadeh, S.; Ghorbani-Asl, M.; Hlawacek, G.; Krasheninnikov, A. V. Supported Two-Dimensional Materials under Ion Irradiation: The Substrate Governs Defect Production. *ACS Appl Mater Interfaces* **2018**, *10* (36), 30827–30836.

[6] Gong, Y.; Liu, Z.; Lupini, A. R.; Shi, G.; Lin, J.; Najmaei, S.; Lin, Z.; Elías, A. L.; Berkdemir, A.; You, G.; Terrones, H.; Terrones, M.; Vajtai, R.; Pantelides, S. T.; Pennycook, S. J.; Lou, J.; Zhou, W.; Ajayan, P. M. Band Gap Engineering and Layer-by-Layer Mapping of Selenium-Doped Molybdenum Disulfide. *Nano Lett* **2013**, *14* (2), 442−449.

[7] Komsa, H.-P.; Krasheninnikov, A. V. Two-Dimensional Transition Metal Dichalcogenide Alloys: Stabilityand Electronic Properties. *J. Phys. Chem. Lett.* **2012**, *3* (23), 3652−3656.
